# Evolutionary rescue in resistance to pesticides

**DOI:** 10.1098/rspb.2024.0805

**Published:** 2024-06-26

**Authors:** Philip G. Madgwick, Thomas Tunstall, Ricardo Kanitz

**Affiliations:** ^1^ Syngenta, Jealott’s Hill International Research Centre, Bracknell RG42 6EY, UK; ^2^ Living Systems Institute, University of Exeter, Exeter EX4 4PY, UK; ^3^ Syngenta Crop Protection, Rosentalstrasse 67, Basel CH-4058, Switzerland

**Keywords:** evolutionary rescue, pesticide resistance, eco-evolutionary dynamics, population genetics, population ecology, resistance management

## Abstract

Evolutionary rescue occurs when the genetic evolution of adaptation saves a population from decline or extinction after environmental change. The evolution of resistance to pesticides is a special scenario of abrupt environmental change, where rescue occurs under (very) strong selection for one or a few de novo resistance mutations of large effect. Here, a population genetic model of evolutionary rescue with density-dependent population change is developed, with a focus on deriving results that are important to resistance management. Massive stochastic simulations are used to generate observations, which are accurately predicted using analytical approximations. Key results include the probability density function for the time to resistance and the probability of population extinction. The distribution of resistance times shows a lag period, a narrow peak and a long tail. Surprisingly, the mean time to resistance can increase with the strength of selection because, if a mutation does not occur early on, then its emergence is delayed by the pesticide reducing the population size. The probability of population extinction shows a sharp transition, in that when extinction is possible, it is also highly likely. Consequently, population suppression and (local) eradication can be theoretically achievable goals, as novel strategies to delay resistance evolution.

## 1. Introduction

Evolutionary rescue describes how a population may survive environmental change that threatens it with decline and/or extinction by the genetic evolution of adaptation to the changed environment [1–3]. This phenomenon has been extensively studied using quantitative genetics in the scenario of biodiversity loss in natural populations due to anthropogenic drivers like pollution, habitat destruction and climate change that lead to a gradually deteriorating environment [2–7]. The other major scenario of evolutionary rescue, which has received less attention [2,8,9], is a single abrupt change in the environment, such as with the application of a pesticide or antibiotic [3]. This scenario differs because pests tend to have specific biological attributes that make them robust to abrupt environmental change, such as large population sizes and fast population growth rates [10], with many of these species having survived previous applications of pesticides [11]. Further, resistance is usually absent in populations prior to pesticide application [12,13], controlled by one or a few genes of large effect [14] and under (very) strong selection [15–18]. Consequently, it is more appropriate to model evolutionary rescue in resistance to a pesticide using classical population genetics (rather than quantitative genetics), where the first step of resistance that rescues the population from decline and/or extinction arises from a single de novo mutation [2,8,9,19,20]. While the adaptive fine-tuning of resistance may then follow the predictions of quantitative genetics, it is the first step of resistance that is key to evolutionary rescue [8,9].

By incorporating both genetic evolution and population change, evolutionary rescue is an eco-evolutionary phenomenon [21]. With the complexity of incorporating genetic evolution and population change into a unified framework, the population genetic models of evolutionary rescue in resistance to pesticides have tended to make a familiar set of simplifying assumptions [2,8,9,19,22]. Following the standard approach of population genetics (from [23]; see also [24,25]), genetic evolution is modelled for haploid individuals with discrete, non-overlapping generations in an unstructured population that is mutation-limited. The standard approach of population ecology models population changes using a growth rate and carrying capacity (from [26,27]; see also [28,29]). To date, for simplicity, models of evolutionary rescue neglect the population carrying capacity by assuming density-independent population growth (as discussed in [8,9]). The resulting models [2,8,9,19,22] are put forward as heuristics, which corroborate the general finding (across models and scenarios) that evolutionary rescue is more likely in larger populations under weaker mortality selection [2,4,5,8,9,20].

An important simplification from the assumption of density-independent population growth is that population extinction can occur under (very) weak mortality selection. Under density-dependent population change, small increases in mortality may drive a population to decline to a new equilibrium rather than extinction, which has unclear effects on the key statistics and distributions that characterize evolutionary rescue. Here, a population genetic model of evolutionary rescue with density-dependent population change is developed. The analysis extends the fundamental theory of the population genetics of evolutionary rescue in response to a single abrupt change in the environment, which has to date been primarily focused on contributing to conservation biology. The model here is focused on the case of resistance to pesticides, with the aim of bridging the gap between existing models of evolutionary rescue and applied models of resistance evolution. For resistance management, the aims are antithetical to conservation biology: rather than focusing on what would protect threatened species from extinction, the aim is to prolong the control of pest species or eradicate them. As such, the analysis characterizes some statistics and distributions that have not been derived previously because of the shift in focus to the goal of delaying and/or preventing evolutionary rescue.

## 2. Methods and results

Following on from the pattern of previous population genetic models of evolutionary rescue (especially in [9]), the aim of the modelling is not just to present results that describe the relevant features of evolutionary rescue in resistance to pesticides for resistance management, but to further explain the key results using analytical derivations and approximations. Consequently, the methods and results are twofold. First, massive simulations of a stochastic model in discrete time are run to generate ‘observations’ that describe evolutionary rescue in resistance to pesticides. The dynamic equations are simplistic, but nonetheless commonly underlie more complicated models of resistance evolution [30–32]. Second, a continuous-time framework is used to generate ‘predictions’ of the key statistics and distributions that characterize the stochastic model. In as far as the predictions are accurate, they help to explain how the parameters drive the results. Continuous-time predictions can accurately approximate the results of those more complicated models that use the same dynamics (as shown in [33]).

### (a) Discrete-time observations

The stochastic model that is used to generate discrete-time observations is based on classic models in population genetics and population ecology, albeit that these models are not classically interpreted in a unified eco-evolutionary model. In keeping with the simplifying assumptions made by its forerunners [8,9,19], these models assume haploid individuals with discrete, non-overlapping generations in an unstructured population that is mutation-limited (i.e. mutations have independent fates). This set-up is selected for its generality and simplicity, but also because it rests on clear biological assumptions [24,25]. Deviations from this set-up are briefly discussed later on (see §2(k)), including using this set-up as a heterozygote approximation for diploid pests [34–36]. The model is described with respect to a single resistance allele in a pest species, whose evolution can be characterized with just four parameters: a population growth rate ( r ), a population carrying capacity ( K ), a resistance allele mutation rate ( μ ) and a selection coefficient ( s ) to describe the relative fitness of resistant-mutant and susceptible-wild-type individuals after pesticidal mortality ( $\omega_{R}=1$ , $\omega_{S}=1/(1+s)$ ). Here, it is convenient to make use of a selection coefficient to describe the relative fitness of resistant and susceptible individuals because it describes fitness with a single parameter (i.e. reducing the number of parameter combinations) and affords the mutant a relative fitness of 1+s in keeping with classical population genetics. Yet, this construction of fitness assumes that there is no fitness cost to resistance (i.e. $\omega_{R}=1$ ); to enable the model to be applied more generally when this assumption does not hold (when $\omega_{R}<1$ ), the model is described in terms of fitness (i.e. $\omega_{R}$ and $\omega_{S}$ ) rather than the selection coefficient ( s ).

The eco-evolutionary model is based on a Wright–Fisher model [37,38] with mutation, mortality selection and an elastic population size. In each generation, there are three sources of stochasticity: mutation, demography and genetic drift. First, allele frequencies are stochastically modified by binomial sampling, where the probability is the mutation rate ( μ ) and the number of trials is the population size ( N ). The mutation rate is the forward rate at which wild-type alleles mutate into the resistance allele per generation, assuming that backward mutation from the rare resistance allele to the wild-type allele is absent. Second, like other models of evolutionary rescue, population size is modulated by mean fitness [2,8]. But unlike other models, population change incorporates rK -type density-dependence of the form that assumes non-overlapping generations ([28,29]; see also electronic supplementary material, table S1 for the form that assumes overlapping generations). In this form, the population carrying capacity ( K ) is the maximum population size in the ecological niche (that is not the same as the equilibrium population size), and the population growth rate ( r ) is the maximum rate of population increase per generation when a population is far below its equilibrium. Based on classical population ecology for single species, the population size in the next generation depends on the population growth rate ( r ) and the population size in relation to the carrying capacity ( K ), but also mean fitness ( $\overset{-}{\omega }$ ) after pesticidal mortality:


(2.1) $$N^{\prime }=N\overset{-}{\omega }r\left(1-\frac{N\overset{-}{\omega }}{K}\right).$$

The substitution of N in the classic model by $N\overset{-}{\omega }$ here assumes that pesticidal mortality takes effect before births and density-dependent mortality. Like mutation, population size is stochastically sampled from a binomial distribution, where the probability is the expectation of survival after pesticidal and density-dependent mortality (and reproduction; $N\overset{-}{\omega }r\left(1-N\overset{-}{\omega }/K\right)/K$ ) and the number of trials is the population carrying capacity ( K ). Third, allele frequencies also change stochastically in response to selection and drift. From classical population genetics [23,24], the selection dynamics for the frequency of a resistant allele in the next generation ( $f_{R}^{\prime }$ ) can be calculated from the fitness of resistant and susceptible individuals ( $\omega_{R}$ , $\omega_{S}$ ) and their frequencies ( $f_{R}$ , $f_{S}$ ):


(2.2) $$f_{R}^{\prime }=\frac{f_{R}\omega_{R}}{f_{R}\omega_{R}+f_{S}\omega_{S}}.$$

Allele frequencies are stochastically modified by binomial sampling, where the probability is the expectation of the post-selection frequency of the resistance allele ( $f_{R}^{\prime }$ ) and the number of trials is the population size (i.e. the stochastically sampled $N^{\prime }$ ). The combined effects of the three sources of stochasticity on population size and resistance allele frequency dynamics are exemplified in electronic supplementary material, figures S1 and S2.

The stochastic dynamics of genetic and population change form a unified eco-evolutionary model, which is used to generate a number of pertinent results that characterize resistance evolution. Each simulation starts with the absence of the resistant allele in a population size at an equilibrium population size ( $K_{0}$ ). Susceptible fitness is assumed to be unity prior to pesticidal mortality (so $\omega_{S}=\overset{-}{\omega }=1$ ). As such, the equilibrium population size can be calculated from the population growth rate ( r ) and the population carrying capacity ( K ):


(2.3) $$K_{0}=K\left(\frac{r-1}{r}\right).$$

At the end of each simulation, the key output that is recorded is the ‘time to resistance’, which is afforded its classic interpretation as the time it takes for a resistant allele to reach 50% frequency in the population [39]. A genetic measure of the time to resistance is generally preferred to a population measure because of its simplicity and comparability. When population extinction occurs, a time to resistance cannot be recorded. The combined outputs of many replicates (i.e. repeated runs of the same parameter combination) can be used to estimate the key results of the probability density function for the time to resistance and the probability of population extinction.

Massive simulations are run to generate results across parameter combinations. For the primary simulation, each parameter combination is run for 5000 generations with 1 00 000 replicates, which is sufficient for estimating probability density functions. Three datasets have been generated for different purposes. The first examines six selection coefficients ( s={0.2,0.4,0.8,2,4,8} ) under standard conditions for the other parameters ( r=2 , $K_{0}=10^{7}$ , $\mu =10^{-8}$ ), which is used for plotting distribution figures in the main text. The standard parameters were chosen to give the pest the highest population growth rate that affords a stable (i.e. not oscillatory or chaotic) equilibrium population size [40], the largest population carrying capacity that could be computed [10], and a low mutation rate that nonetheless rarely prevents resistance evolution from a de novo mutation [12,13]. The second dataset examines 100 selection coefficients ( s={0.05-5} ) under standard conditions for the other parameters ( r=2 , $K_{0}=10^{7}$ , $\mu =10^{-8}$ ), which is used for plotting statistic figures across selection coefficients ( s ) in the main text and electronic supplementary material. The third examines all combinations of six selection coefficients ( s={0.2,0.4,0.8,2,4,8} ), five population growth rates ( r={1.1,1.25,1.5,2,3} ), five equilibrium population sizes ( $K_{0}=\{10^{5},10^{6},10^{7},10^{8},10^{9}\}$ ) and five mutation rates (expressed in the form $\mu K_{0}=\left\{10^{-3},10^{-2},10^{-1},10^{0},10^{1}\right\}$ to avoid nonsensical rates), which is used for exploring the parameter space in the electronic supplementary material. A secondary simulation is run to estimate the probability of spreading, due to computational constraints on its accurate estimation. Each parameter combination is run for 5000 generations with a constant population size of 1000. The simulation tracks the allele frequency distribution after selection and genetic drift (i.e. binomial sampling). The R code and data for all the simulations are available in the electronic supplementary material (electronic supplementary material, files S1 and S2).

### (b) Continuous-time predictions

**Figure 1.**
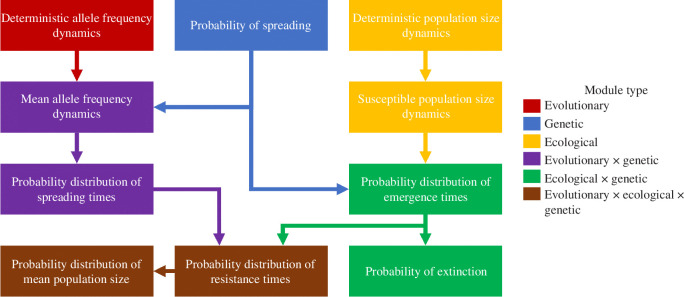
Overview of the modules of the continuous-time framework that is used to generate predictions of the key dynamics, distributions and statistics from discrete-time simulations. Colours show how results are derived by integrating approximations from different module types.

Alongside presenting the observed results from the massive simulations of the stochastic model in discrete time, a continuous-time framework is used to predict the statistics and distributions, including those on route to the key results. The approach to prediction partitions the problem into independent modules (following similar methods in [24]; see also [41]), which can then be integrated together to generate other results. An important strength of the modular approach is that, if one prediction fails or can be improved, then the follow on predictions can be altered without disposing of the framework. An important weakness of the modular approach is that it neglects the interactions between processes that are treated independently. The modules are summarized in figure 1. The ‘evolutionary’ modules build from a continuous-time derivation of the allele frequency dynamics to an approximation of the probability density function of spreading times, which is the time that it takes from a resistance mutation arising to it reaching a frequency threshold (50%). The approximation excludes those resistance mutations that arise but do not spread (because they are lost from the population by genetic drift) by integrating an approximation of the probability of spreading. The ‘ecological’ modules build from a continuous-time derivation of the population size dynamics to an approximation of the probability density function of emergence times, which is the time that it takes for a resistance mutation that will spread (i.e. that escapes loss due to genetic drift) to arise. The approximation factors in those resistance mutations that arise but do not spread, allowing the distributions of spreading and emergence times to be integrated together into a probability density function of resistance times, which is the key result. Another key result is the probability of population extinction, which can be calculated from the probability that a resistance mutation does not emerge. The R code and data for all the predictions are available in the electronic supplementary material (electronic supplementary material, file S3), as well as the figures that are used to compare observed and predicted data (electronic supplementary material, files S4 and S5).

### (c) Probability of spreading

The probability of spread corresponds to the calculation of the classic statistic in population genetics for the probability of fixation, derived using a branching process model as: $1-e^{-2s}\approx 2s$ where s is the selection coefficient ([37,42]; see also [38]). The only salient difference here between the classical approaches and the modern derivation using the diffusion approximation is the clarification that this derivation is only accurate under weak selection [43,44], whereas the evolution of resistance often occurs under strong selection [15–17]. There is little value in a hard definition of what selection coefficients correspond to weak or strong selection in general because any such threshold would depend on the sensitivity of the analysis in question. But, for the sake of clarity here, strong selection is taken to imply s>0.1 , which includes some of the lower mortality rates that are used in the experimental evolution of resistance in the laboratory, and very strong selection is taken to be s>1 , which corresponds to the >50% mortality rates that are expected from pesticides in the field [18].

Three observations are important to extending the approximation under stronger selection. First, the classic result affords a logical construction for the relationship between the selection coefficient and the probability of loss ( $e^{-2s}$ ). Second, the probability of fixation of a neutral mutation is approximately $1/K_{0}$ when $K_{0}$ is the population size [44]. Third, currently, there is no calculation to approximate the probability of an allele’s loss due to drift in each generation after the first in a Wright–Fisher model with selection. The probability of an allele’s loss can nonetheless be conservatively approximated (i.e. underestimated) by the loss in the first generation. Therefore, the probability of spread ( $P_{S}$ ) can be set-up in relation to these fixed points along with some unknown constant ( c ):


(2.4) $$P_{S}\approx 1-\left(E_{1}+ce^{-2s}\right),$$

where $E_{1}={\left(\frac{K_{0}-1}{K_{0}+s}\right)}^{K_{0}}$ is the binomial probability that a mutation is sampled to have 0 frequency in its first generation. The constant ( c ) can be solved using the probability of spread for a neutral mutation: $\pi \approx 1/K_{0}$ , ignoring that population size is not constant, which gives: $c=1-e^{-1}$ . Consequently:


(2.5) $$P_{S}\approx 1-\left({\left(\frac{K_{0}-1}{K_{0}+s}\right)}^{K_{0}}+\left(1-e^{-1}\right)e^{-2s}\right).$$

**Figure 2.**
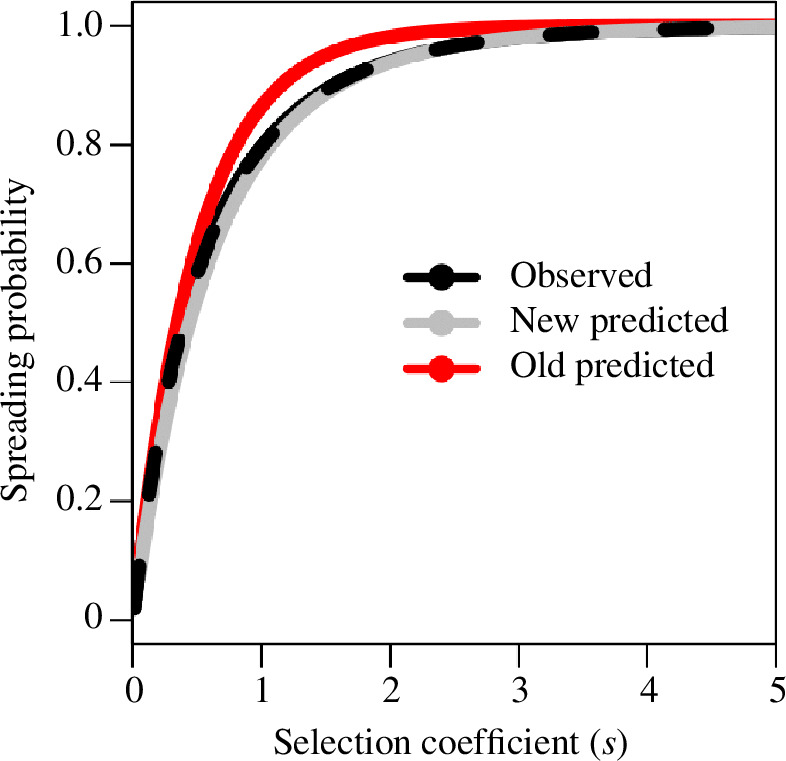
Comparison across selection coefficients of the observed probability of spread in discrete-time simulations (black) and the predicted probability of spread (red) as approximated by the classic expression $1-e^{-2s}$ and as rederived here (grey) in equation (2.5).

This approximation is observed to yield a much better fit than the classic result in a comparable simulation that assumes a constant population size (figure 2).

### (d) Allele frequency dynamics

Building on classical population genetics [23,24], following an established method in resistance modelling [34–36], the discrete-time equation for resistance allele frequency can be reformulated as a continuous-time equation. This can be derived using the discrete-time equation (equation (2.2)), which can be shown by attempting to calculate the allele frequency two generations into future (rather than one):


(2.6) $$f_{R}^{{\;}^{\prime \prime }}=\frac{\left(\frac{f_{R}\omega_{R}}{f_{R}\omega_{R}+f_{S}\omega_{S}}\right)\omega_{R}}{\left(\frac{f_{R}\omega_{R}}{f_{R}\omega_{R}+f_{S}\omega_{S}}\right)\omega_{R}+\left(1-\frac{f_{R}\omega_{R}}{f_{R}\omega_{R}+f_{S}\omega_{S}}\right)\omega_{S}}=\frac{{\left(\omega_{R}/\omega_{S}\right)}^{2}}{{\left(\omega_{R}/\omega_{S}\right)}^{2}+(f_{S}/f_{R})}.$$

The structure of the result suggests a general reformulation with respect to any number of time units t (including non-discrete-time units) from some starting frequencies for the resistant and susceptible alleles ( $f_{R,0}$ , $f_{S,0}$ ):


(2.7) $$f_{R,t}=\frac{{\left(\omega_{R}/\omega_{S}\right)}^{t}}{{\left(\omega_{R}/\omega_{S}\right)}^{t}+\left(f_{S,0}/f_{R,0}\right)}.$$

This calculation is not an approximation, but rather an exact description in as far as fitness can be described without using frequency terms (i.e. for haploidy, without consideration of dominance). The classic derivation [23] takes a further step that relies upon very weak selection (demonstrated with s≈0.001 ) to take a simple approximation (of $f_{R,t}=1/(1+e^{st})$ ), but this is unnecessary with modern computers.

The continuous-time equation for genetic change (equation (2.6)) permits the calculation of the time that it takes for a resistant allele to spread to a frequency threshold ( $f_{T}$ ):


(2.8) $$T_{S}=log\left(f_{T}f_{S,0}/\left(1-f_{T}\right)f_{R,0}\right)/\log\left(\omega_{R}/\omega_{S}\right).$$

This provides a complete description of the expected allele frequency dynamics when they are deterministic following the discrete iterator in equation (2.2)—and is not an approximation.

### (e) Population size dynamics

Building on classical population ecology [28,29], the discrete-time difference equation for resistance allele frequency can be reformulated as a continuous-time equation. The method is well-established [26,27], although there is a slight adjustment to include mean fitness (and its effects) in the derivation. Having already established the dynamic of genetic evolution, the population size dynamics follow on with the calculation of mean fitness with respect to time:


(2.9) $${\overset{-}{\omega }}_{t}=f_{R,t}\omega_{R}+f_{S,t}\omega_{S}.$$

Given mean fitness, the new equilibrium population can be calculated ( $K_{t}$ ) as:


(2.10) $$K_{t}=K\left(\frac{r{\overset{-}{\omega }}_{t}-1}{r{\overset{-}{\omega }}_{t}^{2}}\right).$$

The discrete-time equation for population change can be reformulated as a differential equation in continuous time:


(2.11) $$\frac{dN}{dt}\approx N\prime -N=N_{t}{\overset{-}{\omega }}_{t}r\left(1-\frac{N_{t}{\overset{-}{\omega }}_{t}}{K}\right)-N_{t}.$$

This equation can be rearranged with simplification by substituting in the new population equilibrium ( $K_{t}$ ):


(2.12) $$\frac{1}{N\left(K_{t}-N\right)}.dN\approx \frac{r{\overset{-}{\omega }}_{t}^{2}}{K}.dt.$$

Treating ${\overset{-}{\omega }}_{t}$ and $K_{t}$ as terms, the differential equation for population change can be integrated between $K_{0}$ at time t=0 and N at time t (which is now denoted as $N_{t}$ for clarity):


(2.13) $$\log\left(\frac{N_{t}\left(K_{t}-K_{0}\right)}{K_{0}\left(K_{t}-N_{t}\right)}\right)\approx r\frac{K_{t}}{K}{\overset{-}{\omega }}_{t}^{2}t.$$

The $N_{t}$ terms can then be collected to give the final expression of population size dynamics over time:


(2.14) $$N_{t}\approx \frac{K_{0}K_{t}e^{\left(r{\overset{-}{\omega }}_{t}-1\right)t}}{K_{t}+K_{0}\left(e^{\left(r{\overset{-}{\omega }}_{t}-1\right)t}-1\right)}.$$

This logistic equation follows a classic structure where $K_{0}$ is the starting value, $K_{t}$ is the ending value and $r{\overset{-}{\omega }}_{t}-1$ is the growth rate; it only differs for its classic form [26,27] because $K_{t}$ and $r{\overset{-}{\omega }}_{t}-1$ can modulate the direction of population change. The expression for population size through time is an approximation because ${\overset{-}{\omega }}_{t}$ and $K_{t}$ were treated as terms (rather than functions that vary over time).

The approximation can need additional specifications under rapid population decline. This is logical, given that under density-dependent population change, a declining population eventually approximates density-independent population change (i.e. $N_{t}{\overset{-}{\omega }}_{t}/K\approx 0$ ), which causes the previous calculation (equation (2.14)) to become inaccurate. Above a critical timepoint ( τ ), the population size transitions to a new calculation:


(2.15) $$N_{t}\approx N_{t-\tau }{\left(r{\overset{-}{\omega }}_{t}\right)}^{t-\tau }.$$

This result is similar to previous density-independent models of evolutionary rescue (eqn 1 in [2]; see also the model description in [8,9]). The critical timepoint ( τ ) can be found as the timepoint where the gradient of the population change is equal for both equations:


(2.16) $$\tau \approx \frac{K}{r{\overset{-}{\omega }}_{t}K_{t}}\log\left(\frac{\left(\frac{r{\overset{-}{\omega }}_{t}^{2}K_{t}}{K}-\log\left(r{\overset{-}{\omega }}_{t}\right)\right)\left(K_{t}-K\right)}{K\log\left(r{\overset{-}{\omega }}_{t}\right)}\right).$$

The full expression for population size can then be described as:


(2.17) $$N_{t}\approx \left\{\begin{array}{cc} \frac{KK_{t}e^{\left(r{\overset{-}{\omega }}_{t}-1\right)t}}{K_{t}+K\left(e^{\left(r{\overset{-}{\omega }}_{t}-1\right)t}-1\right)} & t\leq \tau \\ N_{t-\tau }{\left(r{\overset{-}{\omega }}_{t}\right)}^{t-\tau } & t>\tau \end{array}\right\}.$$

This provides a complete approximation of the expected deterministic population size dynamics. This approximation is highly accurate (see electronic supplementary material, figure S3). The number of susceptible or resistant individuals in the population can be calculated by multiplying the population size (equation (2.19)) by the allele frequency (equation (2.7)) for any point in time.

### (f) Distribution of emergence times

Emergence time is the length of time until a resistance mutation arises that will spread through the population. The established solution assumes a constant population size [45], so adjustment must be made for the susceptible population size dynamics. These can be calculated in the same way as the dynamics for the whole population, substituting mean fitness through time with the constant susceptible fitness ( ${\overset{-}{\omega }}_{t}=\omega_{S}$ ). Assuming that there are no resistant individuals ( $f_{R}=0$ ), a new susceptible population equilibrium can be calculated to replace the population equilibrium through time ( $K_{t}=K_{S}$ ; see equation (2.10)):


(2.18) $$K_{S}=K\left(\frac{r\omega_{S}-1}{r\omega_{S}^{2}}\right)=\frac{K\left(\left(r-1\right)-s\right)\left(1+s\right)}{r}.$$

Using this result, the full expression for susceptible population size is then:


(2.19) $$N_{S,t}\approx \left\{\begin{array}{cc} \frac{K_{0}K_{S}e^{\left(r\omega_{S}-1\right)t}}{K_{S}+K_{0}\left(e^{\left(r\omega_{S}-1\right)t}-1\right)} & t\leq \tau \\ N_{S,\tau }{\left(r\omega_{S}\right)}^{t-\tau } & t>\tau \end{array}\right\}.$$

It is also necessary to calculate the cumulative susceptible population size through time. This can be calculated by integration within definite limits of time. The susceptible population size may also need to be adjusted when it exceeds the critical timepoint, which gives:


(2.20) $$\sum N_{S,t}\approx \left\{\begin{array}{cc} {\left[\frac{K_{S}}{r\omega_{S}-1}\log\left(K_{S}+K_{0}\left[e^{\left(r\omega_{S}-1\right)t}-1\right]\right)\right]}_{0}^{t} & t\leq \tau \\ \sum N_{S,\tau }+N_{S,\tau }{\left[\frac{{\left(r\omega_{S}\right)}^{t-t^{*}}}{\log\left(r\omega_{S}\right)}\right]}_{\tau }^{t} & t>\tau \end{array}\right\}.$$

The susceptible population size and its cumulative estimate are the only population estimates that are needed going forward to approximate the time to emergence.

The time to emergence can be calculated using a binomial distribution on the basis of calculating the probability that one or more mutations will arise and spread through the population. This method assumes that the population is mutation limited (i.e. on average less than one mutation per generation) because the probability of a spreading mutation arising is approximated by multiplying the probability of spreading per mutation and the probability of mutation per individual ( $P_{S}\mu$ ). The probability that there are no spreading mutations is the complement of the probability of a spreading mutation, which is sampled as many times as there are susceptible individuals in the population, which is the susceptible population size: ${\left(1-P_{S}\mu \right)}^{N_{S,t}}$ . The probability that there is at least one spreading mutation is the complement of this: $1-{\left(1-P_{S}\mu \right)}^{N_{S,t}}$ . In calculating the probability of a spreading mutation first arising, it is necessary to take into account that no spreading mutation has arisen previously, which can be approximated: ${\left(1-P_{S}\mu \right)}^{\sum N_{S,t-1}}$ . In the first generation of selection, this probability must be corrected to 0, which is already a property of equation (2.20) when interpreted with the definite range between time 0 and t . Assembling these pieces together, the time to emergence is:


(2.21) $$T_{E,t}\approx \left(1-{\left(1-P_{S}\mu \right)}^{N_{S,t}}\right){\left(1-P_{S}\mu \right)}^{\sum N_{S,t-1}}.$$

**Figure 3.**
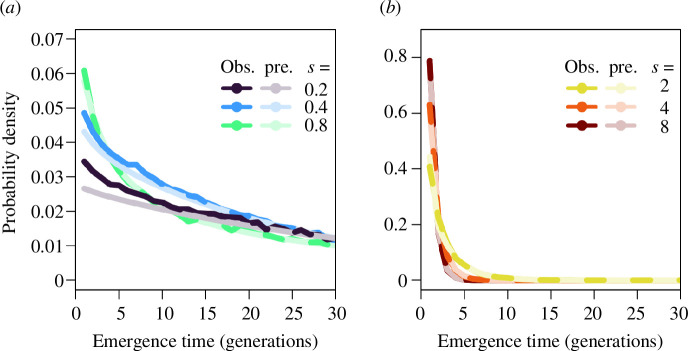
Comparison of the observed probability density function for emergence times in discrete-time simulations (bold colours) and the predicted probability density function for emergence times (faded colours) as approximated in equation (2.21) for (*a*) lower and (*b*) higher selection coefficients.

The distribution has a similar shape to a negative exponential distribution, which is a highly accurate approximation, although it consistently underpredicts the probability of emergence in the first few generations (figure 3; see also electronic supplementary material, figure S4 for a wider parameter space). The mean time to emergence can then be approximated by substituting the susceptible population size with the susceptible population equilibrium ( $N_{S,t}\approx K_{S}$ and $\sum N_{S,t-1}\approx K_{S}t$ ), and integrating for times above time 0 (i.e. to +inf ):


(2.22) $${\overset{-}{T}}_{E}\approx \left|\frac{1-{\left(1-P_{S}\mu \right)}^{K_{S}}}{{\left(K_{S}\log\left(1-P_{S}\mu \right)\right)}^{2}}\right|.$$

This approximation is reasonably accurate (see electronic supplementary material, figure S5). The approximation can be simplified further to $1/K_{S}P_{S}\mu$ when s<r−1 (like eqn 17 in [45]).

### (g) Distribution of spreading times

Spreading time, which is the length of time it takes for a resistance mutation to reach a frequency threshold in the population, depends on the allele frequency dynamics. There is no established solution (except for neutral mutations; see eqn. 2.17 in [46]). The time for a resistant allele to spread is often calculated from an initial frequency that is appreciably rare (e.g. $10^{-5}$ ) to a frequency threshold of 50% ( $f_{T}=½$ ). The allele frequency dynamic (equation (2.7)) and the statistic (equation (2.8)) are highly accurate at predicting the mean case. Under strong selection, the distribution of spreading times is very closely described by a normal distribution around the deterministically estimated mean (equation (2.8)) because genetic drift (captured by binomial sampling) acts equally as a random effect in either direction. The standard deviation is observed to scale with the mean; a Taylor series can be used to expand the mean, which yields an approximation of the form: s.d. = 1/*s*. This result is logical given that a larger selection coefficient means that a wider range of allele frequencies will spread beyond the frequency threshold in a given unit of time.

However, when a resistance allele starts from a single mutation, there is an appreciable chance that the resistant allele will be lost due to chance. Consequently, the relevant ‘time to spread’ that can (in due course) be combined with the ‘time to emergence’ to calculate the ‘time to resistance’ is conditional upon the spread of the resistant allele. The calculation of the mean time to spread conditional upon spread must be corrected to account for the ‘missing probability’ when a resistant allele is lost (as discussed in [9]). There is no accurate approximation of the probability of loss through time, which overwhelmingly occurs when a beneficial mutation has very low copy number. As the effect of the ‘missing probability’ is to increase the rate of spread when it is calculated as conditional upon spread, the most accurate, general approximation to correct for this is simply to divide through by the probability of spread ( $P_{S}$ ; see equation (2.5)):


(2.23) $$f_{R,t}\approx \frac{{\left(\omega_{R}/\omega_{S}\right)}^{t}}{{\left(\omega_{R}/\omega_{S}\right)}^{t}+\left(f_{S,0}/f_{R,0}\right)}/P_{S}$$

This is a conservative approximation that assumes in its calculation that the total probability that the resistant allele is lost due to chance is lost in the first generation (as no more accurate approximation of loss through time could be derived), which would only be the case under selection that is sufficiently strong, such that the allele has a high copy number (that is no longer at risk of loss by genetic drift) after one generation. The continuous-time equation for genetic change (equation (2.23)) permits the calculation of the time that it takes for a resistant allele to spread to a frequency threshold ( $f_{T}$ ):


(2.24) $${\overset{-}{T}}_{S}\approx log\left(P_{S}f_{T}f_{S,0}/\left(1-P_{S}f_{T}\right)f_{R,0}\right)/\log\left(\omega_{R}/\omega_{S}\right).$$

The mean time to spread conditional upon spread is usually estimated to be slightly faster than its deterministic expectation (see equation (2.8)). Again, the distribution of times to spread ( $T_{S,t}$ ) is approximately normal around this mean, with an unaffected standard deviation of s.d. = 1/*s* (or $s.d.=1/\left(\frac{\omega_{R}}{\omega_{S}}-1\right)$ to avoid confusing s and S notation):


(2.25) $$T_{S,t}\approx \frac{\left(\frac{\omega_{R}}{\omega_{S}}-1\right)}{\sqrt{2\pi }}e^{-\frac{1}{2}{\left(\left(\frac{\omega_{R}}{\omega_{S}}-1\right)\left(t-{\overset{-}{T}}_{S}\right)\right)}^{2}}.$$

**Figure 4.**
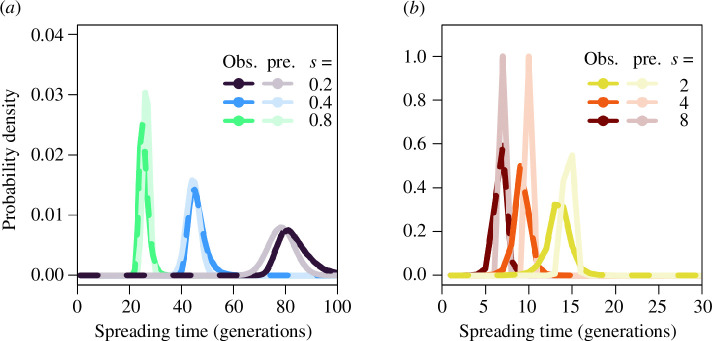
Comparison of the observed probability density function for spreading times in discrete-time simulations (bold colours) and the predicted probability density function for spreading times (faded colours) as approximated in equation (2.25) for (*a*) lower and (*b*) higher selection coefficients.

The overall prediction of the normal distribution is reasonably accurate (figure 4; see also electronic supplementary material, figure S6 for a wider parameter space). The approximation for the standard deviation is observed to provide a good description, while the predicted mean is a slight overestimation across selection coefficients (electronic supplementary material, figure S7). If an allele started with an appreciable starting copy number, then the approximation would be more accurate because the binomial sampling process would equally increase or decrease its frequency relative to its expectation. When an allele starts from a very low copy number, the distribution is asymmetric because of the directional bias that is introduced by an allele that decreases in frequency being more likely to be lost from the population by genetic drift.

### (h) Distribution of times to resistance

There is no analytical expression for the time to resistance, which is the time it takes for a resistance mutation to emerge and spread. Instead, this distribution must be calculated by the convolution of emergence and spreading times, which involves adding together the distributions of all spreading times for an allele starting its spread in each generation, weighted by the probability of starting in that generation from the distribution of emergence times. As the distribution of spreading times is normal (and comparatively narrowly distributed) and the distribution of emergence times appears negative exponential-like (with a broad tail), the distribution of resistance times is often very similar to the distribution of emergence times with an offset from 0 of the mean time to resistance (that represents the lag-time for how long it takes a resistant allele to spread). In some situations, this simpler approximation may be preferable as an analytical approximation of the distribution of times to resistance.

**Figure 5.**
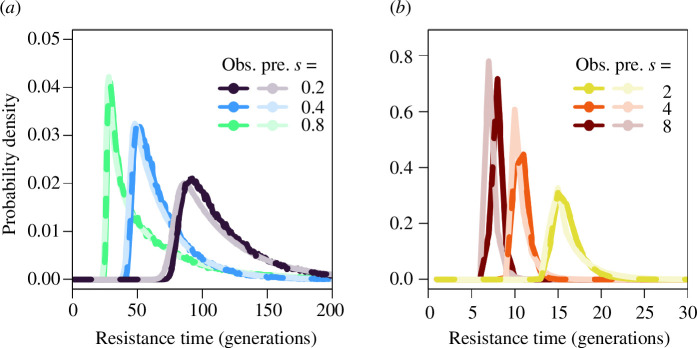
Comparison of the observed probability density function for resistance times in discrete-time simulations (bold colours) and the predicted probability density function for resistance times (faded colours) as approximated by the convolution of the predicted probability density functions for emergence and spreading times for (*a*) lower and (*b*) higher selection coefficients.

The resulting distribution from convolution reveals a positively skewed distribution with an abrupt left-hand side and a long tail on the right-hand side (figure 5; see also electronic supplementary material, figure S8 for a wider parameter space). The comparison across selection coefficients reveals a strong correlation whereby smaller means are associated with smaller standard deviations. In general, there is an increasingly accurate prediction of the distribution of resistance times under stronger selection, but the continuous-time prediction of a discrete-time observation can lead to a discretization error, which can make the prediction become offset from the observation by up to a generation for some higher selection coefficients (e.g. s=8 in figure 5*b*). The shapes of the underlying distributions can be described by key summary statistics that describe the probability density function for the times to resistance. First, as the modal emergence time is always the first generation because emergence times have a negative exponential-like distribution, and the modal spreading time is the same as its mean because spreading times are normally distributed, the modal resistance time is the mean spreading time (electronic supplementary material, figure S7). Second, following similar logic, the mean resistance time is the sum of the means for the emergence and spreading times (electronic supplementary material, figure S9). Finally, because of the shape of the resistance time distribution, the median is extremely likely to fall between the mode and the mean, and is highly likely to be closer to the mean than the mode.

### (i) Distribution of extinction times

If a resistance mutation does not emerge, populations can become extinct. In general, large populations (>10^3^) are extremely unlikely to go extinct from demographic stochasticity, even over a long time. For the application of a pesticide to give an appreciable chance of population extinction, the susceptible population equilibrium with the application of the pesticide must be less than 0 ( $K_{S}$ ; equation 2.18). From solving this limit, population extinction is only appreciably possible when: s≥r-1 (meaning that pesticidal mortality exceeds the population growth rate). It is conceivable that population extinction could occur when s≈r-1 by bringing the population size below that of a large population (< 10^3^), and this limit could be solved (but 10^3^ is not a hard threshold to make this calculation especially informative in a general context).

**Figure 6.**
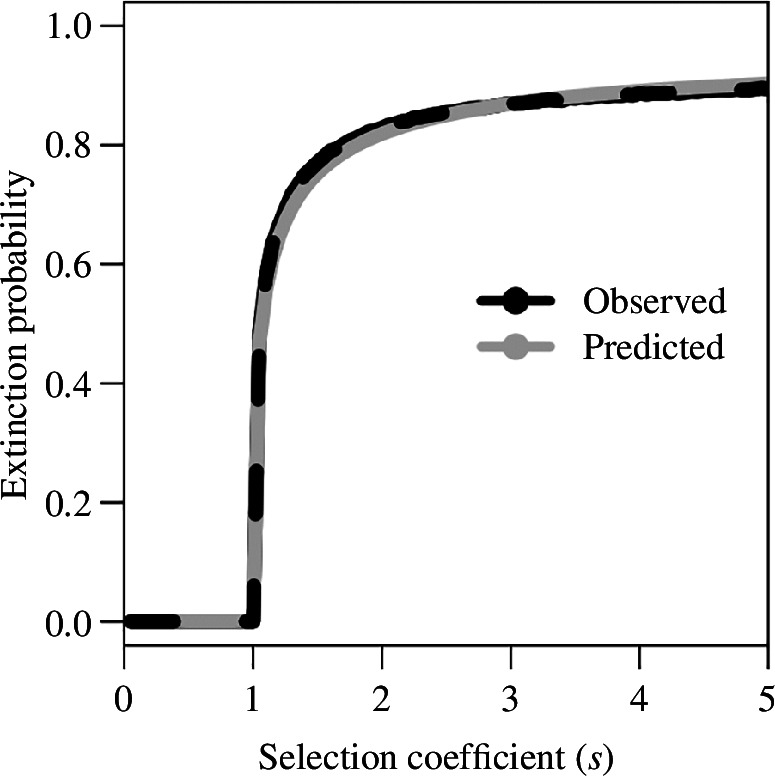
Comparison across selection coefficients of the observed probability of extinction in discrete-time simulations (black) and the predicted probability of extinction (grey) as approximated by $1-\sum T_{E,t}$ .

The probability of extinction can be calculated assuming the initial absence of a resistant mutation. There is no analytical expression for the probability of extinction, but it can be simply calculated as the complement of the sum of the distribution of emergence times ( $1-\sum T_{E,t}$ ; similar to eqn 3 in [8]). An interesting feature of the probability of extinction that emerges from this analysis is that when extinction becomes possible (when s≥r-1 ), it very quickly becomes highly likely as the rate of pesticide mortality increases beyond this limit, which is accurately predicted (figure 6). Also within this parameter range, interestingly, the probability of extinction can explain the shorter long tail of the time to resistance distribution under larger selection coefficients because, if a mutation does not occur early, then the population is liable to go extinct before the mutation can arise at all.

The time to population extinction has a distribution that depends on the susceptible population size. The probability that an individual survives demographic stochasticity can be given as $N_{S,t}/K$ . The probability that it does not survive is the complement ( $1-N_{S,t}/K$ ). For no individuals to survive, the probability of not surviving would need to be sampled by the number of trials, which is the population carrying capacity ( K , leading to: ${\left(1-N_{S,t}/K\right)}^{K}$ ). The probability of extinction at a given time must also take into account that the population cannot have gone extinct at a previous time, which is well approximated by the probability of not surviving in the previous time. Altogether, the distribution of the time to extinction is:


(2.26) $$T_{X,t}\approx {\left(1-\frac{N_{S,t}}{K}\right)}^{K}-{\left(1-\frac{N_{S,t-1}}{K}\right)}^{K}.$$

**Figure 7.**
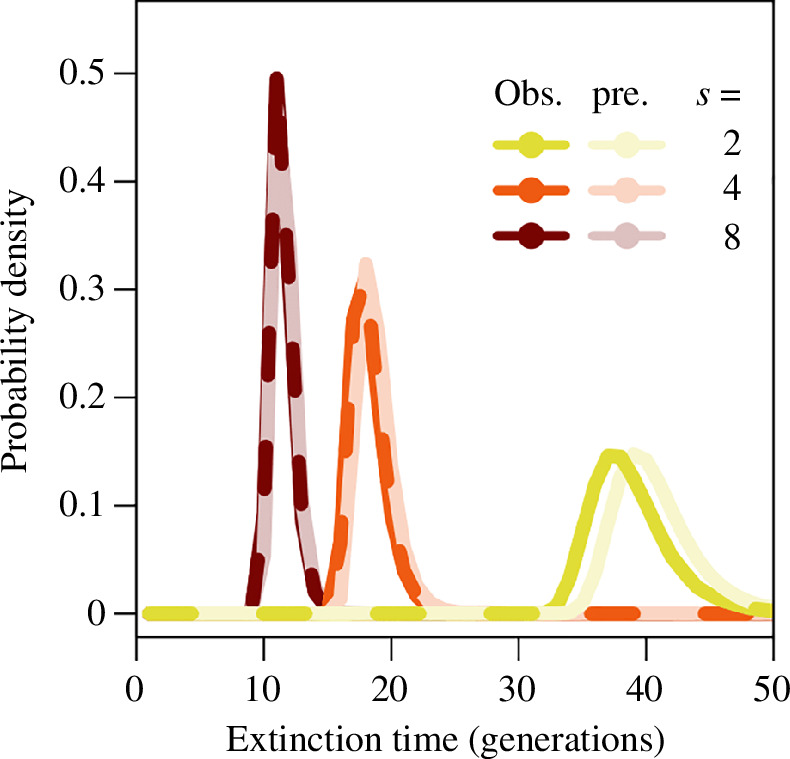
Comparison of the observed probability density function for extinction times in discrete-time simulations (bold colours) and the predicted probability density function for extinction times (faded colours) as approximated in equation (2.26) for higher selection coefficients where extinction is possible.

This is a highly accurate approximation (figure 7; see also electronic supplementary material, figure S10 for a wider parameter space). The distribution is narrow from the mean tendency when there is a large number of trials, as a result of the large carrying capacity.

When extinction is possible, the mean time to extinction can be calculated using the susceptible population size dynamics (equation (2.19)). The susceptible population size dynamics asymptote as they approach 0, and so the expected time to extinction can be solved using the limit of one individual as:


(2.27) $${\overset{-}{T}}_{X}\approx \tau -log\left(N_{S,\tau }\right)/log\left(r\omega_{S}\right).$$

This is also a highly accurate approximation (electronic supplementary material, figure S11). When s≥r-1 , the mean time to extinction (equation (2.27)) falls between the mean (the sum of equations (2.22) and (2.24)) and mode (equation (2.24)) of the time to resistance.

### (j) Distribution of mean population size

A distribution of mean population size can also be formulated. The distribution of the time to resistance is critical to estimating the mean population size in describing the point at which a resistant allele starts to obtain high frequencies. The mean must be taken over a timeframe of interest ( ζ ), which should be taken as larger than the vast majority of times to resistance (but not so large as to make the time without resistance negligible). There are various approaches to calculating results that could be taken. A simple approach assumes that the population is at its susceptible size ( $N_{S,t}$ ) before the time to resistance and returns to its initial equilibrium ( $K_{0}$ ; given that $\omega_{R}=1$ ) after the time to resistance:


(2.28) $${\overset{-}{N}}_{t}\approx \frac{1}{\zeta }\left(T_{R,t}\frac{1}{\zeta }\sum_{0}^{\zeta }N_{S,t}+\left(\zeta -T_{R,t}\right)K_{0}\right).$$

Although this approximation ignores non-equilibrium population size dynamics after the time to resistance, it is reasonably accurate, especially for lower selection coefficients when population extinction does not occur (electronic supplementary material, figure S12); for higher selection coefficients, alternative measures like the probability of population extinction may be more appropriate anyway.

### (k) Extensions for violated assumptions

The continuous-time framework has assumed haploid individuals with discrete, non-overlapping generations in an unstructured population that is mutation-limited. With careful consideration, the framework can be applied to make predictions when these assumptions are violated. First, the framework can be extended to diploids. Most easily, as has been well-established [34–36], the haploid predictions may be taken as a heterozygote approximation, with dominance captured in the set-up of fitness and appropriate adjustment to the probability of spreading [43,44]. The heterozygote approximation is highly accurate because a resistant allele is overwhelmingly found within heterozygotes as it arises and spreads from rarity. Alternative extensions to diploidy are possible [20,30]. Second, the model can be extended to continuous, overlapping generations by rederiving the model using appropriate discrete-time equations for allele frequency and density-dependent population change (see electronic supplementary material, table S1). An alternative approach is to piece together this result by using the discrete-time equations to describe independent cohorts [47]. Third, the continuous-time framework can flex to incorporate structured populations by providing a focused description of genetic and population change in subpopulations. The derivations are agnostic to the whole-population structure. If subpopulation movements are expected to introduce resistant individuals, migration can be incorporated as part of the mutation rate [24]. If the population growth rate ( r ) varies between subpopulations, subpopulation extinction is more likely in less favourable environments with lower population growth rates (see [30] for a discussion). Fourth, environmental stochasticity can be incorporated into the model by sampling population parameters ( r , K ) from a distribution in each generation ([48]; see also [49]). Such variation may introduce noise into the population dynamics that cannot be predicted by the continuous-time framework, but this is not expected to substantially alter the key distributions of emergence, spreading and resistance times that have been derived from the allele frequency dynamics (based on ongoing work by the authors). Finally, a population that is not mutation-limited is highly likely to have resistant individuals present at a neutral equilibrium, giving the resistant allele a starting frequency. Without an emergence time, the time to resistance simplifies to equate the mean spreading time because more common alleles behave increasingly deterministically [2,9]. Therefore, the continuous-time framework is expected to be robust to many violations of its assumptions; the details of any other violations that break the predictions may be of interest for future research.

## 3. Discussion

To explore evolutionary rescue in resistance to pesticides, massive simulations of a stochastic model in discrete time have been run to provide observations and a continuous-time framework has been used to explain the key statistics and distributions by prediction. The model extends the population genetic theory of evolutionary rescue in response to a single abrupt change in the environment by incorporating density-dependent population change. The analysis also shifts the focus of existing theory from conservation biology to resistance management, deriving results that have been previously neglected.

One such key result is the probability density function for the time to resistance (figure 5), which shows the distribution of times to evolutionary rescue. The distribution has a lag period determined by the length of time it takes for a resistance allele to spread through the population, a narrow peak around the averages for the time to resistance (with the mode first, the mean last and the median in between, closer towards the mean) and a long tail. The distribution of resistance times changes peculiarly as the strength of selection increases, which is reflected in the non-monotonic relationship with the mean resistance time (electronic supplementary material, figure S10). The pattern of the non-monotonic relationship arises from the change in the mean emergence time (electronic supplementary material, figure S5), which describes the average wait time until a resistance mutation arises that will spread through the population. Surprisingly, the mean emergence time can increase with the strength of selection because, if a mutation does not emerge earlier, then its emergence is delayed by the reduction in the population size (that makes a resistance mutation less likely to occur). Consequently, the complicated relationship is a result of the density-dependent population change, which is absent from other population genetic models of evolutionary rescue.

Another important derivation is the probability of population extinction that other studies have previously examined [2,8,19], but again it is also surprisingly complicated by density-dependent population change. When the strength of selection becomes strong enough to make population extinction a possibility, it also becomes highly likely (figure 6). The sharp transition occurs because the threshold for the possibility of extinction coincides with the mean extinction time becoming less than the mean resistance time (electronic supplementary material, figures S9 and S11).

For resistance management, the results explain the surprising possibility of win–win scenarios where more effective population control can also delay or prevent the evolution of resistance (that is also observed but not explained in [30–32]). Models of resistance evolution have tended to focus on the comparison of the mean spreading time for resistance alleles with appreciable initial frequency [39], where greater population control always leads to a shorter time to resistance (as shown in electronic supplementary material, figure S7). This neglects the emergence time that it takes for a resistance mutation to arise that will spread through the population, which may be especially important in the evolution of resistance to new pesticides. The emergence time can be delayed by suppressing the population size through more effective population control or prevented by even more effective population control driving the population to extinction. Indeed, the sharp transition to high levels of population extinction suggests that eradication is a theoretically achievable goal (at least, in a locality), as a strategy to delay resistance. In this way, by incorporating emergence time into considerations, suppression and eradication may join moderation, saturation and redundancy as the principles of resistance management, describing independent mechanisms of counteracting resistance evolution [41,50].

## Data Availability

The R scripts that were used to generate the discrete-time observations, the continuous-time predictions and all the figures in the main text and the electronic supplementary material are available on Dryad [51]. Supplementary material is available online [52].
